# Real‐world outcomes for high‐risk non‐muscle‐invasive bladder cancer: screened patients for the BRAVO trial

**DOI:** 10.1111/bju.16516

**Published:** 2024-09-26

**Authors:** Samantha Conroy, Ibrahim Jubber, Aidan P. Noon, Derek J. Rosario, Jon Griffin, Susan Morgan, Rachel Hubbard, Steve Kennish, Stephen Mitchell, Suresh Venugopal, Kate Linton, Ramanan Rajasundaram, Syed A. Hussain, James W.F. Catto

**Affiliations:** ^1^ Division of Clinical Medicine University of Sheffield Sheffield UK; ^2^ Department of Urology Sheffield Teaching Hospitals NHS Foundation Trust Sheffield UK; ^3^ Department of Histopathology Sheffield Teaching Hospitals NHS Foundation Trust Sheffield UK; ^4^ Department of Radiology Sheffield Teaching Hospitals NHS Foundation Trust Sheffield UK; ^5^ Department of Urology, Wycombe Hospital Buckinghamshire Healthcare NHS Trust High Wycombe UK; ^6^ Department of Urology The Royal Liverpool and Broadgreen University Hospitals Liverpool UK; ^7^ Department of Urology Chesterfield Royal Hospital NHS Foundation Trust Chesterfield UK; ^8^ Department of Urology Doncaster Royal Infirmary Doncaster UK; ^9^ Department of Oncology, Weston Park Hospital Sheffield Teaching Hospitals NHS Foundation Trust Sheffield UK

**Keywords:** High‐risk non‐muscle‐invasive bladder cancer, BCG, radical cystectomy, feasibility study, bladder cancer, real‐world data, surgical trial, RCT

## Abstract

**Objective:**

To report real‐world outcomes for high‐risk non‐muscle‐invasive bladder cancer (HRNMIBC), including bacillus Calmette‐Guérin (BCG) and radical cystectomy (RC), as randomised comparisons of these have not been possible.

**Methods:**

We detail consecutive participants screened for the BRAVO randomised controlled trial comparing RC with BCG (International Standard Randomised Controlled Trial Number [ISRCTN]12509361). Patients were prospectively registered and case‐note review used for outcomes. The primary outcome was overall survival. Secondary outcomes included recurrence, progression, metastasis, and bladder cancer‐specific survival.

**Results and limitations:**

A total of 193 patients were screened, including 106 (54.9%) who received BCG, 43 (22.3%) primary RC, 37 (19.2%) ‘other’ treatment and seven (3.6%) hyperthermic intravesical mitomycin C. All‐cause death occurred in 55 (28.5%) patients at median (interquartile range [IQR]) of 29.0 (19.5–42.0) months. In multivariable analysis, overall mortality was more common in older patients (hazard ratio [HR] 2.63, 95% confidence interval [CI] 1.35–5.13; Cox *P* = 0.004 for age >70 years), those recruited from district hospitals (HR 0.53, 95% CI 0.3–0.95; *P* = 0.032) and those who did not undergo RC as their first treatment (HR 2.16, 95% CI 1.17–3.99; *P* = 0.014). In all, 17 (8.8%) patients died from bladder cancer (BC) at median (IQR) of 22.5 (19–36.25) months. In multivariable analysis, BC‐specific mortality was more common in older patients (HR 4.87, 95% CI 1.1–21.6; *P* = 0.037) and those with Tis/T1 disease (HR 2.26, 95% CI 1.23–4.16; *P* = 0.008) but did not vary with initial treatment.

**Conclusions:**

Patients with HRNMIBC are at high‐risk of mortality. Those choosing RC as their initial treatment have lower risks of mortality than others, although this may reflect fitness and selection.

**Abbreviations**
BC: bladder cancer
CIS: carcinoma in situ
CONSORT: Consolidated Standards of Reporting Trials
CSM: cancer‐specific mortality
CSS: cancer‐specific survival
HIVEC: hyperthermic intravesical mitomycin C
HR: hazard ratio
HR: high risk
IQR: interquartile range
MDT: multidisciplinary team
MFS: metastasis‐free survival
(N)MIBC: (non‐)muscle‐invasive bladder cancer
OS: overall survival
PFS: progression‐free survival
QPI: quality performance indicator
RC: radical cystectomy
RFS: recurrence‐free survival
RWD: real‐world data
TURBT: transurethral resection of bladder tumour

## Introduction

Bladder cancer (BC) is a common malignancy [1]. In England, >18 000 people are diagnosed every year, of which ~70% are non‐muscle‐invasive BCs (NMIBCs) [2]. NMIBC is heterogenous in nature, where tumour grade, stage, presence of carcinoma *in situ* (CIS), histological subtype, size, location and multiplicity contribute to behaviour [3, 4]. Patients with high‐risk NMIBC (HRNMIBC) are commonly managed using transurethral resection of bladder tumour (TURBT) with adjuvant intravesical BCG [4]. BCG offers bladder preservation but fails to control the disease in the majority of patients [5] and up to one third do not complete maintenance treatment [6]. Primary radical cystectomy (RC) is an alternative approach, which removes the bladder and may minimise the risk of recurrence and progression. However, RC is overtreatment for non‐progressing tumours and is a major undertaking [7, 8] for this comorbid population [9], with long‐term quality‐of‐life implications [10, 11].

Randomised controlled trials (RCTs) are necessary to understand the relative risks and benefits of BCG and RC. Given differences in modality, a phase III RCT has not been undertaken to date. To understand if this was possible, we conducted the BRAVO feasibility study, in which we randomised patients with HRNMIBC to receive either intravesical BCG or RC [12]. Recruitment was challenging and so we concluded a full phase III RCT comparing these options was unlikely to fully recruit in an affordable timeframe.

An alternative to randomised trials for comparing treatments, is the use of real‐world data (RWD) [13]. RWD are less expensive to collect, may better reflect the actual clinical environments in which patients are treated and are more inclusive of differing demographics and comorbidity status. For reliable RWD, populations should be comparable and data collection robust. Screening logs curated during trial recruitment, as described in the Consolidated Standards of Reporting Trials (CONSORT) statement, may provide a valuable resource [14]. First, in providing insight into reasons for non‐recruitment [15] and second, as a prospective cohort of eligible patients who better reflect the real‐world populations we treat in the clinic. Hence, although RWD does not provide randomised and controlled comparisons, it can provide an adjunct to RCT data, particularly where clinical trials are logistically or financially challenging to perform.

In the present study, we describe the outcomes of the prospectively screened cohort for the BRAVO trial. This represents a potentially homogenous population of patients with HRNMIBC, as defined by eligibility for an RCT.

## Methods

### Participants and Study Approvals

Consecutive participants were identified from the South Yorkshire and Bassetlaw Cancer Network screening logs (April 2016–March 2018). All cases were identified from the regional cancer multidisciplinary team (MDT) meeting (Sheffield Teaching Hospitals NHS Foundation Trust). Patient demographics, eligibility and randomisation status were prospectively collected. Retrospective case note review was performed to determine endpoints (finalised: 28 June 2023). BRAVO was a multicentre, parallel‐group, mixed‐methods, individually randomised, controlled feasibility study within seven recruiting cancer networks [12, 16]. The trial received ethical approval from the Yorkshire and Humber National Research Ethics Service Committee (16/YH/0268) and sponsored by Sheffield Teaching Hospitals NHS Trust (International Standard Randomised Controlled Trial Number [ISRCTN]12509361).

### Eligibility Criteria

In this study, we included all consecutive patients screened for eligibility for the BRAVO clinical trial in the South Yorkshire region, which included 193 patients from the 407 screened across all sites. BRAVO eligibility criteria have been described previously [16]. Not all screened patients were eligible or randomised within the BRAVO clinical trial, but all were initially screened on the basis of having a suspected non‐muscle‐invasive bladder tumour with high‐grade features.

### Endpoints

Our primary outcome was overall survival (OS). Secondary outcomes included recurrence‐free survival (RFS) in patients whose first treatment was a bladder preserving, and progression‐free survival (PFS), metastasis‐free survival (MFS), and cancer‐specific mortality (CSM) for the whole cohort. The 2‐ and 5‐year RFS, PFS, MFS, cancer‐specific survival (CSS), and OS proportions were yielded with 95% CIs from Kaplan–Meier plots, with the exception of patients first treated with hyperthermic intravesical mitomycin C (HIVEC) (seven patients), for whom 5‐year outcomes were not reported. Recurrence was defined as histologically confirmed intravesical disease for patients with their bladder *in situ*. Progression was a stage increase for those with their bladder *in situ* (e.g. Ta to T1 or Ta–1 to ≥T2). Patients receiving RC, or ‘other’ treatment were removed from recurrence and progression survival analyses, as they either had no bladder or it was uncertain if they were included in a cystoscopic surveillance programme to accurately detect and/or sample recurrences. Metastases were identified from imaging (mostly CT scan) as disease outside of the bladder (locoregional or distant metastases). Deaths were identified from hospital records/cancer registries, and cause defined by consensus using medical records and death certification. Consensus reviewers (S.C., D.J.R. and J.W.F.C.) were blinded to demographics, histology, treatment, and treatment centre to minimise bias. Follow‐up duration and time to event was calculated as time passed from initial histological diagnosis in months.

### Quality Performance Indicators

Quality performance indicators (QPIs) minimise variance in clinical practice and adherence to minimum standards may improve clinical outcomes [17, 18, 19]. The following QPIs were relevant to HRNMIBC: QPI1—MDT discussion (target: 95%); QPI2—quality of TURBT (documentation, complete resection, detrusor muscle included); QPI4—early re‐TURBT (target: 80% within 42 days [in this study, local target was within 3 months of initial TURBT]); QPI11—30/90 day mortality after treatment (target: <5%); and QPI12–clinical trial and research access. All patients in this cohort were discussed at the regional network MDT (QPI1). Operating note documentation (QPI2) was not available for two‐thirds of the cohort (as they were outside of Sheffield). Clinical trial access (QPI12) was achieved. We have therefore focussed on completeness of resection, detrusor muscle sampling, re‐TURBT rate and interval from initial TURBT, as well as, 30/90 day mortality.

### Statistical Analysis

No power calculation was performed as this was an observational cohort study. Continuous variables (age, times to event) are described using median with interquartile ranges (IQRs), and categorical variables (all others) as counts and percentages. Comparisons were performed using chi‐squared, Fisher's exact or Mann–Whitney *U* tests, according to variable. Uni‐ and multivariable survival analyses were performed using Cox regression for recurrence, progression, and death (all cause and BC‐specific) with age >70 years, male sex, randomisation status, treatment site, any ≥T1 histopathology, any CIS, re‐resection status and first treatment. Hazard ratios (HRs) are presented with 95% CIs. The Kaplan–Meier method was used to plot survival. All tests were two‐sided and statistical significance was defined as a *P* < 0.05. Analyses were performed using the Statistical Package for the Social Sciences (SPSS®), version 29 (IBM Corp., Armonk, NY, USA ) and GraphPad Prism, version 10.0.3 (GraphPad Software Inc., San Diego, CA, USA).

## Results

### Patients, Tumours and Treatments

**Fig. 1:**
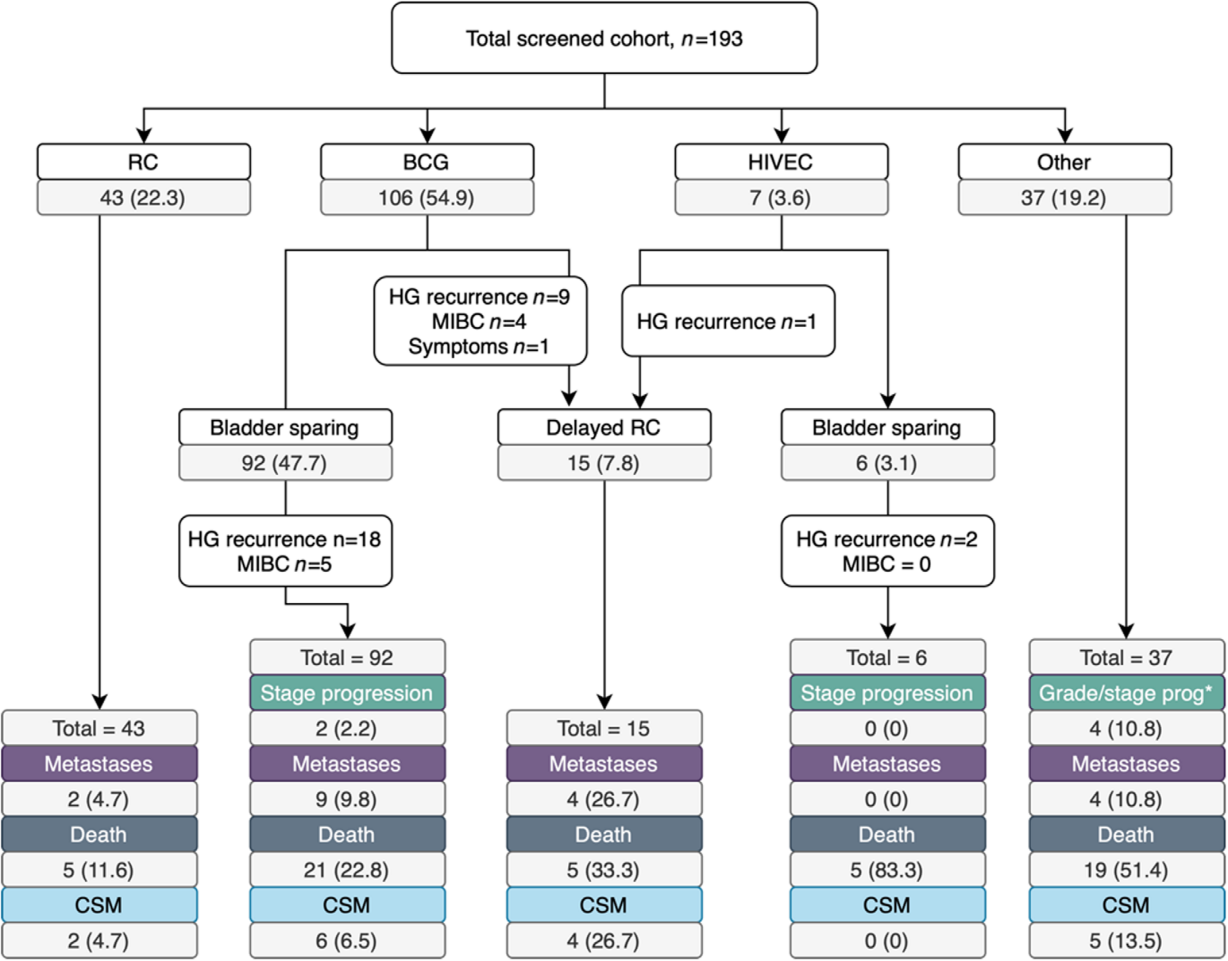
Patient flow in BRAVO screened cohort. The CONSORT diagram details first and subsequent treatment, as well as progression, metastases, and deaths per group. *Two patients progressed from low‐grade to high‐grade (HG) tumours.

Our population included 193 patients screened for eligibility for the BRAVO clinical trial [12] (Table 1). The median (IQR) age was 72.0 (66.5–79.0) years, 154 (79.8%) patients were male and most diagnosed at district hospitals (*n* = 125 [64.8%]). The median (IQR) follow‐up was 55 (46.0–60.5) months. After screening, 46 (23.8%) participants were randomised into BRAVO; this included 27 patients randomised to BCG and 19 patients randomised to primary RC. Across the whole cohort, most patients received BCG (*n* = 106 patients [54.9%]; randomised within BRAVO *n* = 27 [25.5%]) as their first treatment (Fig. 1 and Table 2), followed by primary RC (*n* = 43 [22.3%]; randomised within BRAVO *n* = 19 [44.2%]), ‘other’ (*n* = 37 [19.2%] including surveillance or watchful waiting) and HIVEC (seven patients [3.6%]). Features associated with treatment choice included age, randomisation status, hospital type, grade, presence of histological subtype and re‐resection status (Tables 1 and 2). The randomised cohort was younger (median [IQR] age 70.0 [63.8–77.0] vs 74.0 [67.0–80.0] years; Mann–Whitney *U P* = 0.009) and more likely to have been diagnosed at the cancer centre (rate: 47.8% vs 31.3%, chi‐squared *P* = 0.041) than the non‐randomised cohort.

**Table 1:** Patient and tumour characteristics for the screening log cohort stratified by recruitment into the BRAVO RCT.

Characteristic	Not recruited (*n* = 147)	Randomised into BRAVO (*n* = 46)
Age, years, median (IQR)	74.0 (67.0–80.0)	70.0 (63.8–77.0)
Age (years), *n/N* (%)		
0–70	53/77 (68.8)	24/77 (31.2)
≥71	94/116 (81.0)	22/116 (19.0)
Sex, *n/N* (%)		
Female	30/9 (76.9)	9/39 (23.1)
Male	117/154 (76.0)	37/154 (24.0)
Hospital type, *n/N* (%)		
Cancer centre	46/68 (67.6)	22/68 (32.4)
District hospital	101/125 (80.8)	24/125 (19.2)
Grade, *n/N* (%)		
2	34/41 (82.9)	7/41 (17.1)
3	107/144 (74.3)	37/144 (25.7)
Stage, *n/N* (%)		
Tis	5/7 (71.4)	2/7 (28.6)
Ta	76/100 (76.0)	24/100 (24.0)
T1	61/81 (75.3)	20/81 (24.7)
T2	3/3 (100.0)	0/3 (0.0)
Tx	2/2 (100.0)	0/2 (0.0)
Growth, *n/N* (%)		
Papillary	99/124 (79.8)	25/124 (20.2)
Mixed	27/40 (67.5)	13/40 (32.5)
Solid	4/7 (57.1)	3/7 (42.9)
Background urothelium, *n/N* (%)		
Normal	26/32 (81.3)	6/32 (18.8)
Dysplasia	6/10 (60.0)	4/10 (40.0)
CIS	51/74 (68.9)	23/74 (31.1)
Muscle in TURBT specimen, *n/N* (%)		
No	42/49 (85.7)	7/49 (14.3)
Yes	105/144 (72.9)	39/144 (27.1)
Histological subtype, *n/N* (%)		
No	133/175 (76.0)	42/175 (24.0)
Yes	14/18 (77.8)	4/18 (22.2)

**Table 2:** Initial treatments received after diagnosis.

Characteristic	BCG (*n* = 106)	HIVEC (*n* = 7)	RC (*n* = 43)	Other (*n* = 37)	Chi sq. *P*
Age, years, median (IQR)	72.0 (66.0–78.3)	75.0 (71.0–81.0)	71.0 (65.0–77.0)	77.0 (69.0–81.5)	**0.033** *
Age (years), *n/N* (%)					
0–70	45/77 (58.4)	1/77 (1.3)	20/77 (26.0)	11/77 (14.3)	0.205
≥71	61/116 (52.6)	6/116 (5.2)	23/116 (19.8)	26/116 (22.4)	
Sex, *n/N* (%)					
Female	21/39 (53.8)	1/39 (2.6)	10/29 (25.6)	7/39 (17.9)	0.93
Male	85/154 (55.2)	6/154 (3.9)	33/154 (21.4)	30/154 (19.5)	
Randomised into BRAVO, *n/N* (%)					
No	79/147 (53.7)	7/147 (4.8)	24/147 (16.3)	37/147 (25.2)	**<0.001**
Randomised	27/46 (58.7)	0/46 (0.0)	19/46 (41.3)	0/46 (0.0)	
Background urothelium sampled, *n/N* (%)					
No	43/77 (55.8)	6/77 (7.8)	12/77 (15.6)	16/77 (20.8)	**0.031**
Yes	63/116 (54.3)	1/116 (0.9)	31/116 (26.7)	21/116 (18.1)	
Hospital type, *n/N* (%)					
Cancer centre	32/68 (47.1)	7/68 (10.3)	16/68 (23.5)	13/68 (19.1)	**0.003**
District	74/125 (59.2)	0/125 (0.0)	27/125 (21.6)	24/125 (19.2)	
Grade, *n/N* (%)					
2	20/41 (48.8)	3/41 (7.3)	5/41 (12.2)	13/41 (31.7)	**0.027**
3	81/144 (56.3)	4/144 (2.8)	37/144 (25.7)	22/144 (15.3)	
Background urothelium, *n/N* (%)					
Normal	18/32 (56.3)	1/32 (3.1)	6/32 (18.8)	7/32 (21.9)	0.385
Dysplasia	7/10 (70.0)	0/10 (0.0)	1/10 (10.0)	2/10 (20.0)	
CIS	38/74 (51.4)	0/74 (0.0)	24/74 (32.4)	12/74 (16.2)	
Stage, *n/N* (%)					
Tis	4/7 (57.1)	0/7 (0.0)	1/7 (14.3)	2/7 (28.6)	0.39
Ta	52/100 (52.0)	7/100 (7.0)	21/100 (21.0)	20/100 (20.0)	
T1	48/81 (59.3)	0/81 (0.0)	19/81 (23.5)	14/81 (17.3)	
T2	1/3 (33.3)	0/3 (0.0)	2/3 (66.7)	0/3 (0.0)	
Tx	1/2 (50.0)	0/2 (0.0)	0/2 (0.0)	1/2 (50.0)	
Histological subtype, *n/N* (%)					
No	101/175 (57.7)	7/175 (4.0)	36/175 (20.6)	31/175 (17.7)	**0.048**
Yes	5/18 (27.8)	0/18 (0.0)	7/18 (38.9)	6/18 (33.3)	
Re‐TURBT, *n/N* (%)					
No	27/62 (43.5)	5/62 (8.1)	14/62 (22.6)	16/62 (25.8)	**0.027**
Yes	79/131 (60.3)	2/131 (1.5)	29/131 (22.1)	21/131 (16.0)	

Bold values statistically significant at *P* < 0.05.

* Kruskal–Wallis.

### Quality Performance Indicators

In total, 144 (74.6%) patients had muscle in their initial TURBT specimen and 131 (67.9%) underwent re‐TURBT. Muscle was more likely to be present in younger patients (83.1% for ≥70 years vs 69.0%, Chi‐squared *P* = 0.027), with T1 disease (84% vs 68% for Ta and 71% Tis, *P* = 0.011) and in specimens that included background urothelium (81.9% with vs 63.6% without urothelium, *P* = 0.004, Table S1). Of those who underwent re‐TURBT, 57 (43.5%) had residual disease (52 high‐grade and five low‐grade tumours) and up‐staging occurred in seven (5.3%) patients, two (1.5%) to muscle invasion. Of the 62 who did not have a re‐TURBT, 40 (64.5%) had Ta disease with or without CIS and 31 (50.0%) had sufficient muscle sampled. Two (3.2%) patients had T2 disease in their initial specimen. Although the care of randomised patients was more compliant with the QPIs, the differences did not reach statistical significance (Table S2).

### Outcomes from Bladder Preserving Approaches

**Fig. 2:**
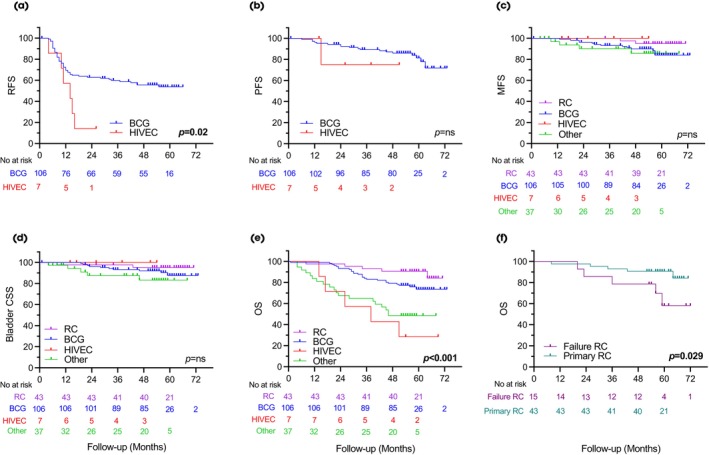
Survival outcomes stratified by initial treatment received. Kaplan–Meier survival analysis detailing (**a**) local recurrence in patients receiving bladder‐sparing treatments (including BCG and HIVEC, (**b**) progression to more advanced disease for bladder‐sparing treatments, (**c**) MFS, (**d**) Bladder CSS, (**e**) OS, and (**f**) OS for patients receiving primary or treatment‐failure RC. Univariable Cox regression (two‐sided) *P* values are shown.

Bladder preserving approaches were the first management strategy for 150 (77.2%) patients. Of these, 63 (41.3%) patients experienced recurrence (median [IQR] time of 11 [7–16.3] months). In multivariable analysis (Table S3a), factors associated with recurrence included sex (male: HR 0.27, 95% CI 0.1–0.71; Cox *P* = 0.008), recruiting hospital (district: HR 0.39, 95% CI 0.16–0.97; Cox *P* = 0.043) and randomisation status (randomised: HR 0.20, 95% CI 0.06–0.72; Cox *P* = 0.014). A total of 27 (18.0%) patients who received bladder preservation progressed, including nine (6.0%) to MIBC and 17 (11.3%) to metastatic BC. RC was used in 15 patients for treatment failure (see section 3.4 below). In multivariable analysis, there were no significant associations between progression events and available parameters (Table S3b). Figure 2a,b displays the RFS and PFS analysis for patients who received BCG and HIVEC therapy. The 2‐year RFS and PFS for patients initially treated with BCG were 62.9% (95% CI 52.9–71.3%) and 92.3% (95% CI 85.2–96.1%), whereas the 5‐year RFS and PFS were 55.8% (95% CI 43.4–64.2%) and 81.3% (95% CI 70.2–88.6), respectively.

### Outcomes from RC

A total of 58 (30.1%) of patients underwent RC, including 43 as primary treatment and 15 for salvage after bladder preservation (Table S4). The median (IQR) times to primary RC and treatment‐failure RC were 4 (3–5) and 13 (10–21) months, respectively (Mann–Whitney *U*, *P* < 0.001). There was no difference in the proportion of patients with T0, ≥T2 histology, residual cancer or upstaging at RC (all chi‐squared *P* > 0.05). In patients who underwent salvage RC, 13 (86.7%) had residual tumour, five (33.3%) had MIBC, and one (6.7%) had N1 disease. Within the primary and salvage RC populations, five patients died during follow‐up from each (11.6% and 33.3%, respectively; Fig. 1). Overall, but not CSS, was significantly poorer in patients undergoing RC for treatment failure (log rank *P* = 0.029, Fig. 2f).

### Progression and Metastases

Progression occurred in 29 (15.0%) patients at a median (IQR) of 24 (14–42) months. In univariable analysis (Table S5 and Fig. 2b), features associated with progression included older age (HR 3.83, 95% CI 1.46–10.04; Cox *P* = 0.006 for age ≥71 years), stage (HR 0.23, 95% CI 0.06–0.082; Cox *P* = 0.024 for Ta disease), and primary treatment (HR 0.22, 95% CI 0.05–0.95; Cox *P* = 0.043 for RC). None of these features were associated with progression in multivariable analysis. Radiologically defined metastases were seen in 19 (9.8%) patients at a median (IQR) of 32 (14–42) months. Seven (36.8%) patients developed locoregional and 12 (63.2%) distant metastases. Metastases were more common in older patients (HR 4.36, 95% CI 1.27–15.00; Cox *P* = 0.019 for age ≥71 years) and with stage Tis/T1 disease (HR 10.09; 95% CI 1.65–61.60; Cox *P* = 0.012; Table S6 and Fig. 2c). In multivariable analysis, only stage remained significantly associated with metastases (HR 8.02, 95% CI 1.22–52.96; Cox *P* = 0.031). The 2‐ and 5‐year MFS proportions were 96.2% (95% CI 90.1–98.5%) and 84.0% (95% CI 73.2–90.7%) for BCG, 100% and 95.0% (95% CI 81.5–98.7%) for RC, and 90.2% (95% CI 72.5–96.7%) and 85.7% (95% CI 65.7–94.4%) for other treatment, respectively.

### Bladder CSS

At reporting, 17 (8.8%) patients had died from BC (median [IQR] of 22.5 [19–36.25] months after treatment). Bladder CSM was more common in older patients (HR 4.87; 95% CI 1.1–21.6; Cox *P* = 0.037 for age ≥71 years) and those with Tis/T1 disease (HR 2.26, 95% CI 1.23–4.16; Cox *P* = 0.008; Table S7 and Fig. 2d). Bladder CSS did not vary by initial treatment choice (HR 2.15, 95% CI 0.77–1.71; Cox *P* = 0.49), although only two patients who underwent primary RC died from the disease (Figs 1 and 2d). The 2‐ and 5‐year bladder CSS values were 96.2% (95% CI 90.1–98.5%) and 87.6% (95% CI 76.7–93.5%) for BCG, 97.7% (95% CI 84.6–99.5%) and 95.2% (95% CI 82.2–98.7%) for RC, and 87.5% (95%CI 70.0–95.1%) and 83.2% (95% CI 63.6–92.6%) for other treatment, respectively.

### Overall Survival

Death from any cause occurred in 55 (28.5%) patients at a median (IQR) of 29.0 (19.5–42.0) months. In those who survived, the median (IQR) follow‐up was 57.0 (54.0–62.0) months. Overall mortality was more common in older patients (HR 2.63, 95% CI 1.35–5.13; Cox *P* = 0.004 for age ≥71 years), those initially recruited from district hospitals (HR 0.53, 95% CI 0.3–0.95; Cox *P* = 0.032) and those who did not chose RC as their first treatment (HR 2.16, 95% CI 1.17–3.99; Cox *P* = 0.014, Fig. 2e, Table 3). The 2‐ and 5‐year OS proportions were 93.4% (95% CI 86.6–96.8%) and 73.6% (95% CI 65.6–84.1%) for BCG, 97.7% (95% CI 84.6–99.6%) and 90.7% (95% CI 77.1–96.4%) for RC, and 67.6% (95% CI 50.0–80.1%) and 48.6% (95% CI 32.0–63.5%) for other treatment, respectively.

**Table 3:** Overall (all cause) mortality within the cohort.

Characteristic	Overall mortality		Univariable Cox		Multivariable Cox	
	*n/N* (%)	95% CI	HR (95% CI)	*P*	HR (95% CI)	*P*
Age (years)						
0–70	11/77 (14.3)	7.8–23.4		**<0.001**		**0.004**
≥71	44/116 (37.9)	29.5–47	3.08 (1.59–5.97)		2.63 (1.35–5.13)	
Sex						
Female	12/39 (30.8)	18–46.2		0.697		
Male	43/154 (27.9)	21.3–35.4	0.88 (0.46–1.67)			
Randomised into BRAVO						
No	50/147 (34.0)	26.7–41.9		**0.005**		0.061
Randomised	5/46 (10.9)	4.3–22.2	0.27 (0.11–0.67)		0.39 (0.15–1.05)	
Hospital type						
Cancer centre	26/68 (38.2)	27.4–50.1		**0.028**		**0.032**
District	29/125 (23.2)	16.5–31.2	0.55 (0.33–0.94)		0.53 (0.3–0.95)	
Grade						
2	11/41 (26.8)	15.2–41.6		0.923		
3	40/144 (27.8)	21–35.5	1.03 (0.53–2.02)			
Background urothelium						
Normal	10/32 (31.3)	17.3–48.4		0.732		
Dysplasia	2/10 (20.0)	–				
CIS	19/74 (25.7)	16.8–36.4	0.61 (0.13–2.77)			
Stage						
Tis	4/7 (57.1)	23.5–86.1		0.46		
Ta	28/100 (28.0)	19.9–37.3				
T1	22/81 (27.2)	18.4–37.5				
T2	0/3 (0.0)	‐				
Tx	1/2 (50.0)	6.1–93.9	0.85 (0.55–1.31)			
Growth						
Papillary	37/124 (29.8)	22.3–38.3		0.978		
Mixed	9/40 (22.5)	11.8–37.1				
Solid	2/7 (28.6)	6.5–64.8	0.98 (0.24–4.07)			
Histological subtype						
No	51/175 (29.1)	22.8–36.2		0.659		
Yes	4/18 (22.2)	8–44.6	0.80 (0.29–2.2)			
Re‐TURBT						
No	22/62 (35.5)	24.5–47.8		0.102		
Yes	33/131 (25.2)	18.4–33.1	0.64 (0.37–1.09)			
First treatment						
BCG	26/106 (24.5)	17.1–33.3		**<0.001**		**0.014**
HIVEC	5/7 (71.4)	35.2–93.5				
RC	5/43 (11.6)	4.6–23.6				
Other	19/37 (51.4)	35.7–66.8	2.82 (1.56–5.1)		2.16 (1.17–3.99)	

Bold values statistically significant at *P* < 0.05.

## Discussion

We present real‐world, long‐term outcomes from the prospectively screened HRNMIBC cohort identified for the BRAVO clinical trial [16]. We highlight that robust data can be yielded from prospective screening logs of clinical trials, which may better reflect the clinical environments and patient cohorts that are treated in clinic, with less bias than other retrospective observational studies. Managing patients with HRNMIBC is an evolving challenge, especially considering a comorbid BC population [9]. Urologists and patients must carefully weigh up the natural history of this cancer, the morbidity of repeated or radical treatments, and outcomes from primary/salvage treatments [20, 21]. Given the challenges with recruitment into BRAVO [12], and the direction of travel within the community (with newer options for NMIBC [22] and the use of bladder‐sparing treatments), it is unlikely that prospective randomised evidence will guide treatment choices.

There are several key findings to discuss. First, most patients (77.2%) initially chose bladder‐preserving approaches, despite known risks of local failure. Recurrences were less likely in males (as reported [23]), randomised patients and in those diagnosed at a district hospital. In patients receiving bladder preservation, progression occurred in 18.0% (risks did not differ by bladder‐preservation strategy) and over one in 10 developed metastases. Salvage RC was used in the minority, and OS outcomes were poorer than with primary RC (but not CSS). Relatively (two patients [13.3%]) few of those undergoing salvage RC were randomised and so there is potential selection bias in this cohort (perhaps less fit patients initially chose BCG and then needed salvage treatments).

Second, quality of care was high (as shown by compliance with QPIs), although trial participants faired marginally better (with QPIs and lower local recurrence rates) than those who were not recruited. It is known that participants in clinical trials fare better than others, through improved surveillance, scheduling and rigour [24]. The re‐TURBT rate was modest (67.9%) and might reflect either a decision not re‐resect in the presence of Ta disease and adequate detrusor sampling (as per European Association of Urology [EAU] guidelines [4]), patient frailty or comorbidity, or patient preference. The emerging role of multiparametric MRI staging coupled with Vesical Imaging‐Reporting and Data System (VI‐RADS) [25] reporting may somewhat alter the way we surgically stage patients in future.

Third, 28.5% of our cohort died by the time of reporting, although few deaths were from BC (8.8%). There was discordance between factors associated with overall (age, hospital and initial treatment) and CSM (age and stage). This conflict suggests differences in OS might reflect patient/surgeon selection (e.g., higher mortality in older patients), rather than treatment effectiveness, although our design precludes certainty. Differences between hospital centres might reflect selection bias (all participants screened in cancer centres vs selective referrals from district hospitals) or more efficient care in smaller units (in which patients with HRNMIBC might be a higher priority). It is perhaps surprising that bladder CSS rates were not different for RC and BCG, given the nature of these two treatments, although a large observational registry cohort of very‐HRNMIBC has again recently suggested equivocal outcomes between these modalities in well‐selected patients [26, 27]. The survival plot (Fig. 2c) shows this mainly reflects the low event rate, although small differences might be present. Thus, a larger study, longer follow‐up or higher‐risk cases (to increase the low event rate) might be needed to fully test the comparison. Regardless, these data are useful for guiding choice withing routine clinical practice and reassure bladder‐preserving strategies (especially given emerging treatments [28, 29].

Fourth, older age was a risk factor for all‐cause mortality and CSM, conflicting with findings from a recent large retrospective study [30], in which older patients (aged >70 years) treated with adequate BCG had non‐inferior CSM to younger patients. The differences in these studies highlight demographic discrepancies, retrospective design and that many (over one‐fifth) of our older patients (aged >70 years) received non‐BCG approaches; nonetheless, highlights the real‐world age discrepancies in the treatments we deliver in the clinic.

There are several limitations to discuss. As with all RWD, selection is common, with treatment allocated by clinician bias, local service provision and patient choice, rather than oncological risk. Confounding data (such as performance status and comorbidity index) and detailed treatment information (such as number/date of intravesical therapy instillations) were not available for many patients, leading to a loss of data granularity. Key event rates (metastases and cancer‐specific deaths) were low, meaning that discernible differences between groups were challenging to identify and multivariable analysis results must therefore be interpreted with caution. Finally, this study population included all patients screened for the BRAVO clinical trial, included those who were eligible and recruited, eligible and not recruited, and those ineligible for the study; hence, also consecutive and non‐discriminatory, there is heterogeneity within the screened cohort, including patients with MIBC. In addition, the cohort only reflects patients reviewed and treated in South Yorkshire (albeit the largest recruiting region for the BRAVO trial).

Nonetheless, the data presented from this reproducible, prospectively screened cohort provides us with evidence to support the use of both bladder‐sparing and radical approaches in patients with HRNMIBC. We can counsel patients with the knowledge that metastases and cancer‐specific deaths in the first 5 years after diagnosis remain low, regardless of treatment received. Longer‐term, prospective follow‐up studies will continue to contribute to our understanding of the natural history of HRNMIBC treated with bladder‐preservation and RC.

## Conclusions

This study highlights the utility of real‐world data collections when using prospectively screened HRNMIBC cohorts. Long‐term outcome data provides useful information for counselling patients, in particular, that metastatic and cancer‐specific death events remain low regardless of treatment, although the risk of all‐cause mortality can be high.

## Author Contributions

Samantha Conroy and James W.F. Catto had full access to all the data in this study and take responsibility for the integrity of the data and the accuracy of the data analysis. Concept and design: Samantha Conroy, Aidan P. Noon, Derek J. Rosario, Jon Griffin, Syed A. Hussain, James W.F. Catto. Acquisition, analysis, or interpretation of data: Samantha Conroy, Jon Griffin, James W.F. Catto. Drafting of the manuscript: All authors. Intellectual content: All authors. Obtained funding: Samantha Conroy, James W.F. Catto. Administrative, technical, or material support: Samantha Conroy, James W.F. Catto.

## Disclosure of Interests

James W.F. Catto has received consulting fees from AstraZeneca, Ferring, Ipsen, Roche, and Janssen; has received speaker fees from Bristol Myers Squibb, Pfizer, Merck Sharp and Dohme, Janssen, Astellas, Nucleix, InMed, and Roche; has received honoraria for membership in advisory boards from Ferring, Roche, Gilead, Photocure, Pfizer, Bristol Myers Squibb, QED Therapeutics, and Janssen; and has received research funding from Roche. Syed A. Hussain has received personal fees AstraZeneca, Merck, Roche, Bristol Myers Squibb, and Janssen; and has received personal fees and grants from Boehringer Ingelheim and Pierre Fabre. Samantha Conroy and Ibrahim Jubber have received speaker fees from InMed. Stephen Mitchell is the clinical lead for urology for Healthcare Business Solutions (HBS) UK.

## Supporting information


**Table S1.** Features associated with the presence of muscle in the TURBT specimen (part of QPI2).
**Table S2.** Compliance with Quality performance indicators within those randomised and not‐randomised into BRAVO.
**Table S3a.** Recurrence within the cohort treated by initial bladder sparing approaches.
**Table S3b.** Progression within the cohort treated by initial bladder sparing approaches.
**Table S4.** Comparative outcomes of patients undergoing RC for primary treatment or RC after primary treatment.
**Table S5.** Progression to more advanced disease within the entire cohort.
**Table S6.** Development of metastases within the entire cohort.
**Table S7.** Bladder CSM within the cohort.
