# Supplementary material for: Real‐world outcomes for high‐risk non‐muscle‐invasive bladder cancer: screened patients for the BRAVO trial
Source: BJU Int. 2024 Sep 26;135(2):329–38. doi: 10.1111/bju.16516 (PMC11745995; doi:10.1111/bju.16516)
Supplement: Supplementary file 1 — Table S1. Features associated with the presence of muscle in the TURBT specimen (part of QPI2). Table S2. Compliance with Quality performance indicators within those randomised and not‐randomised into BRAVO. Table S3a. Recurrence within the cohort treated by initial bladder sparing approaches. Table S3b. Progression within the cohort treated by initial bladder sparing approaches. Table S4. Comparative outcomes of patients undergoing RC for primary treatment or RC after primary treatment. Table S5. Progression to more advanced disease within the entire cohort. Table S6. Development of metastases within the entire cohort. Table S7. Bladder CSM within the cohort. [file BJU-135-329-s001.docx]

**Real word high-risk non-muscle invasive bladder cancer: Outcomes from the consecutive screened cohort for the BRAVO randomised feasibility trial comparing radical cystectomy with BCG immunotherapy**

Supplementary files

|  |  | No muscle  (n=49) | | | Muscle  (n=144) | | |  |
| --- | --- | --- | --- | --- | --- | --- | --- | --- |
|  |  | N | % | 95% CI | N | % | 95% CI | Chi sq. p |
| Age (years) | 0-70 | 13 | 17% | 9.8-26.4 | 64 | 83% | 73.6-90.2 |  |
|  | ≥71 | 36 | 31% | 23.2-39.8 | 80 | 69% | 60.2-76.8 | **0.027** |
| Sex | Female | 9 | 23% | 12.1-37.9 | 30 | 77% | 62.1-87.9 |  |
|  | Male | 40 | 26% | 19.5-33.3 | 114 | 74% | 66.7-80.5 | 0.71 |
| Randomised into BRAVO | No | 42 | 29% | 21.7-36.2 | 105 | 71% | 63.8-78.3 |  |
|  | Randomised | 7 | 15% | 7.1-27.6 | 39 | 85% | 72.4-92.9 |  |
| Hospital type | Cancer centre | 21 | 31% | 20.9-42.5 | 47 | 69% | 57.5-79.1 |  |
|  | District | 28 | 22% | 15.8-30.3 | 97 | 78% | 69.7-84.2 | 0.196 |
| Grade | 2 | 10 | 24% | 13.3-39 | 31 | 76% | 61-86.7 |  |
|  | 3 | 37 | 26% | 19.1-33.3 | 107 | 74% | 66.7-80.9 | 0.866 |
| Background urothelium | Normal | 6 | 19% | 8.2-34.6 | 26 | 81% | 65.4-91.8 |  |
|  | Dysplasia | 0 | 0% |  | 10 | 100% |  |  |
|  | CIS | 15 | 20% | 12.4-30.5 | 59 | 80% | 69.5-87.6 | 0.293 |
| Stage | pTis | 2 | 29% | 6.5-64.8 | 5 | 71% | 35.2-93.5 |  |
|  | pTa | 32 | 32% | 23.5-41.6 | 68 | 68% | 58.4-76.5 |  |
|  | pT1 | 13 | 16% | 9.3-25.2 | 68 | 84% | 74.8-90.7 |  |
|  | pT2 | 0 | 0% |  | 3 | 100% |  |  |
|  | pTx | 2 | 100% |  | 0 | 0% |  | **0.011** |
| Growth | Papillary | 36 | 29% | 21.6-37.4 | 88 | 71% | 62.6-78.4 |  |
|  | Mixed | 9 | 23% | 11.8-37.1 | 31 | 78% | 62.9-88.2 |  |
|  | Solid | 1 | 14% |  | 6 | 86% |  | 0.536 |
| Histologic subtype | No | 47 | 27% | 20.7-33.8 | 128 | 73% | 66.2-79.3 |  |
|  | Yes | 2 | 11% | 2.4-31.1 | 16 | 89% | 68.9-97.6 | 0.144 |
| Reresection | No | 13 | 21% | 12.3-32.3 | 49 | 79% | 67.7-87.7 |  |
|  | Yes | 36 | 28% | 20.4-35.6 | 95 | 73% | 64.4-79.6 | 0.332 |
| Flat urothelium present | No | 28 | 36% | 26.3-47.5 | 49 | 64% | 52.5-73.7 |  |
|  | Yes | 21 | 18% | 11.9-25.8 | 95 | 82% | 74.2-88.1 | **0.004** |

**Supplementary Table 1: Features associated with the presence of muscle in the TURBT specimen (part of QPI2).**

|  | | Randomised  (n=46) | | Not randomised  (n=147) | |  |
| --- | --- | --- | --- | --- | --- | --- |
|  | | N | % | N | % | Chi sq. p |
| **QPI1** - MDT discussion (target: 95%) | | 46 | 100 | 147 | 100 | NA |
| **QPI2** - quality of transurethral resection of bladder tumour (detrusor muscle included in initial TUR specimen); | | 39 | 84.8 | 105 | 71.4 | 0.07 |
| **QPI4** – early re-TURBT (target: 80% within 42 days*); | Performed (any time) | 35 | 76.1 | 96 | 65.3 | 0.17 |
|  | Early re-resect | 34 | 97.1 | 94 | 97.9 | 0.9 |
| **QPI11** - 30/90 day mortality after treatment (target: <5%); | | 0 | 0 | 1 | 0.7 | 0.9 |
| **QPI12** – clinical trial and research access. | | 46 | 100 | 147 | 100 | NA |

* Local target within 3 months

**Supplementary Table 2: Compliance with Quality performance indicators within those randomised and not-randomised into BRAVO.**

|  |  | Recurrence | | | Univariable Cox | | |  | Multivariable Cox | | |  |
| --- | --- | --- | --- | --- | --- | --- | --- | --- | --- | --- | --- | --- |
|  |  | n / total | % | 95% CI | HR | Lower | Upper | p | HR | Lower | Upper | p |
| Age (years) | 0-70 | 24 / 57 | 42.1 | 29.9-55 |  |  |  |  |  |  |  |  |
|  | ≥71 | 39 / 93 | 41.9 | 32.3-52.1 | 1.14 | 0.69 | 1.90 | 0.61 |  |  |  |  |
| Sex | Female | 18 / 29 | 62.1 | 44-77.9 |  |  |  |  |  |  |  |  |
|  | Male | 45 / 121 | 37.2 | 29-46 | 0.52 | 0.30 | 0.90 | **0.02** | 0.27 | 0.10 | 0.71 | **0.008** |
| Randomised into BRAVO | No | 55 / 123 | 44.7 | 36.1-53.5 |  |  |  |  |  |  |  |  |
|  | Randomised | 8 / 27 | 29.6 | 15.1-48.2 | 0.54 | 0.26 | 1.14 | 0.106 | 0.20 | 0.06 | 0.72 | **0.014** |
| Hospital type | Cancer centre | 28 / 52 | 53.8 | 40.4-66.9 |  |  |  |  |  |  |  |  |
|  | District | 35 / 98 | 35.7 | 26.8-45.5 | 0.55 | 0.34 | 0.91 | **0.02** | 0.39 | 0.16 | 0.97 | **0.043** |
| Grade | 2 | 16 / 36 | 44.4 | 29.2-60.6 |  |  |  |  |  |  |  |  |
|  | 3 | 43 / 107 | 40.2 | 31.3-49.6 | 0.93 | 0.52 | 1.65 | 0.806 |  |  |  |  |
| Background urothelium | Normal | 12 / 26 | 46.2 | 28.2-64.9 |  |  |  |  |  |  |  |  |
|  | Dysplasia | 3 / 9 | 33.3 | 10.4-65.2 |  |  |  |  |  |  |  |  |
|  | CIS | 19 / 50 | 38.0 | 25.5-51.8 | 0.56 | 0.16 | 2.00 | 0.375 |  |  |  |  |
| Stage | pTis | 4 / 6 | 66.7 | 28.6-92.3 |  |  |  |  |  |  |  |  |
|  | pTa | 34 / 79 | 43.0 | 32.5-54 |  |  |  |  |  |  |  |  |
|  | pT1 | 23 / 62 | 37.1 | 25.9-49.5 |  |  |  |  |  |  |  |  |
|  | pT2 | 1 / 1 | 100.0 | - |  |  |  |  |  |  |  |  |
|  | pTx | 1 / 2 | 50.0 | 6.1-93.9 | 15.23 | 1.41 | 164.55 | **0.025** | 9.90 | 0.66 | 149.33 | 0.098 |
| Growth | Papillary | 46 / 103 | 44.7 | 35.3-54.3 |  |  |  |  |  |  |  |  |
|  | Mixed | 11 / 29 | 37.9 | 22.1-56 |  |  |  |  |  |  |  |  |
|  | Solid | 1 / 3 | 33.3 | 3.9-82.3 | 0.86 | 0.12 | 6.23 | 0.881 |  |  |  |  |
| Histologic subtype | No | 62 / 139 | 44.6 | 36.5-52.9 |  |  |  |  |  |  |  |  |
|  | Yes | 1 / 11 | 9.1 | 1-35.3 | 0.19 | 0.03 | 1.36 | 0.098 |  |  |  |  |
| Re-resection | No | 20 / 48 | 41.7 | 28.5-55.8 |  |  |  |  |  |  |  |  |
|  | Yes | 43 / 102 | 42.2 | 32.9-51.8 | 0.97 | 0.57 | 1.65 | 0.906 |  |  |  |  |
| First treatment | BCG | 47 / 106 | 44.3 | 35.1-53.8 |  |  |  |  |  |  |  |  |
|  | HIVEC | 6 / 7 | 85.7 | 49.9-98.4 |  |  |  |  |  |  |  |  |
|  | RC * | NA | 0.0 | - |  |  |  |  |  |  |  |  |
|  | Other | 10 / 37 | 27.0 | 14.8-42.7 | 2.80 | 1.18 | 6.62 | **0.019** | 0.30 | 0.08 | 1.21 | 0.091 |
| * Cases with RC as first treatment are excluded | | | | | | | | | | | | |

**Supplementary Table 3a. Recurrence within the cohort treated by initial bladder sparing approaches.**

|  |  | Progression | | | Univariable Cox | | |  | Multivariable Cox | | |  |
| --- | --- | --- | --- | --- | --- | --- | --- | --- | --- | --- | --- | --- |
|  |  | n / total | % | 95% CI | HR | Lower | Upper | p | HR | Lower | Upper | p |
| Age >70yrs | 0-70 | 4 / 57 | 7.0% | 2.4-15.8 |  |  |  |  |  |  |  |  |
|  | ≥71 | 23 / 91 | 25.3% | 17.2-34.9 | 4.35 | 1.50 | 12.59 | **0.007** | 9.25 | 0.90 | 95.20 | 0.061 |
| Sex | Female | 5 / 29 | 17.2% | 6.9-33.7 |  |  |  |  |  |  |  |  |
|  | Male | 22 / 119 | 18.5% | 12.3-26.2 | 1.05 | 0.40 | 2.78 | 0.918 |  |  |  |  |
| Randomised into BRAVO | No | 22 / 121 | 18.2% | 12.1-25.8 |  |  |  |  |  |  |  |  |
|  | Randomised | 5 / 27 | 18.5% | 7.4-35.9 | 0.82 | 0.31 | 2.16 | 0.681 |  |  |  |  |
| Hospital type | Cancer centre | 13 / 50 | 26.0% | 15.4-39.3 |  |  |  |  |  |  |  |  |
|  | District | 14 / 98 | 14.3% | 8.4-22.2 | 0.49 | 0.23 | 1.04 | 0.063 |  |  |  |  |
| Grade | 2 | 4 / 35 | 11.4% | 4-24.9 |  |  |  |  |  |  |  |  |
|  | 3 | 20 / 106 | 18.9% | 12.3-27.1 | 1.60 | 0.55 | 4.70 | 0.39 |  |  |  |  |
| Background urothelium | Normal | 3 / 26 | 11.5% | 3.4-27.7 |  |  |  |  |  |  |  |  |
|  | Dysplasia | 0 / 9 | 0.0% | - |  |  |  |  |  |  |  |  |
|  | CIS | 14 / 50 | 28.0% | 17-41.4 | 2.43 | 0.70 | 8.47 | 0.163 |  |  |  |  |
| Stage | pTis | 3 / 6 | 50.0% | 16.7-83.3 |  |  |  |  |  |  |  |  |
|  | pTa | 10 / 77 | 13.0% | 6.9-21.8 |  |  |  |  |  |  |  |  |
|  | pT1 | 14 / 62 | 22.6% | 13.6-34.1 |  |  |  |  |  |  |  |  |
|  | pT2 | 0 / 1 | 0.0% | - |  |  |  |  |  |  |  |  |
|  | pTx | 0 / 2 | 0.0% | - | 0.23 | 0.06 | 0.85 | **0.027** | 2.19 | 0.4 | 12.12 | 0.37 |
| Growth | Papillary | 17 / 101 | 16.8% | 10.5-25 |  |  |  |  |  |  |  |  |
|  | Mixed | 7 / 29 | 24.1% | 11.5-41.6 |  |  |  |  |  |  |  |  |
|  | Solid | 0 / 3 | 0.0% | - | 1.43 | 0.59 | 3.45 | 0.428 |  |  |  |  |
| Variant pathology | No | 25 / 137 | 18.2% | 12.5-25.3 |  |  |  |  |  |  |  |  |
|  | Yes | 2 / 11 | 18.2% | 4-46.7 | 1.19 | 0.28 | 5.03 | 0.816 |  |  |  |  |
| Re-resection | No | 8 / 46 | 17.4% | 8.6-30.2 |  |  |  |  |  |  |  |  |
|  | Yes | 19 / 102 | 18.6% | 12-27 | 0.93 | 0.41 | 2.12 | 0.854 |  |  |  |  |
| First treatment | BCG | 18 / 106 | 17.0% | 10.8-25 |  |  |  |  |  |  |  |  |
|  | HIVEC | 1 / 5 | 20.0% | 2.3-62.9 |  |  |  |  |  |  |  |  |
|  | RC * | 0 / 0 | 0.0% | - | NA |  |  |  | NA |  |  |  |
|  | Other | 8 / 37 | 21.6% | 10.8-36.7 | 1.84 | 0.80 | 4.26 | 0.153 |  |  |  |  |
| * Cases with RC as first treatment are excluded | | |  |  |  |  |  |  |  |  |  |  |

**Supplementary Table 3b. Progression within the cohort treated by initial bladder sparing approaches.**

|  | RC primary treatment  (n=43 (22.2%)) | RC after treatment  (n=15 (7.8%)) | P value |
| --- | --- | --- | --- |
| Median (IQR) time to RC (months) | 4 (3-5) | 13 (10-21) | **<0.0001** |
| Indication MIBC | 2 (4.7) | 4 (26.7) | **0.034** |
| pT0 | 10 (23.2) | 2 (13.3) | 0.71 |
| Residual cancer | 33 (76.7) | 13 (86.7) | 0.71 |
| Upstaged (any) | 6 (14.0) | 6 (40.0) | 0.059 |
| ≥pT2 in RC specimen | 5 (11.6) | 5 (33.3) | 0.11 |
| N+ at RC | 0 (0)* | 2 (13.3)** | 0.064 |
| Significant prostate cancer*** | 8 (18.6) | 4 (26.7) | 0.49 |

*IQR – interquartile range; RC – radical cystectomy; MIBC – muscle invasive bladder cancer; N+ - node positive disease.*

** nodal dissection performed in 27/43 (62.8%) patients*

*** nodal dissection performed in 5/15 (33.3%) patients*

**** Gleason Grade >3+3=6*

**Supplementary Table 4: Comparative outcomes of patients undergoing RC for primary treatment or RC after primary treatment.**

|  |  | Progression | | | Univariable Cox | | |  | Multivariable Cox | | |  |
| --- | --- | --- | --- | --- | --- | --- | --- | --- | --- | --- | --- | --- |
|  |  | n / total | % | 95% CI | HR | Lower | Upper | p | HR | Lower | Upper | p |
| Age (years) | 0-70 | 5 / 77 | 6.5% | 2.5-13.6 |  |  |  |  |  |  |  |  |
|  | ≥71 | 24 / 114 | 21.1% | 14.4-29.2 | 3.83 | 1.46 | 10.04 | **0.006** | 4.05 | 0.78 | 21.04 | 0.096 |
| Sex | Female | 5 / 39 | 12.8% | 5.1-25.8 |  |  |  |  |  |  |  |  |
|  | Male | 24 / 152 | 15.8% | 10.7-22.2 | 1.26 | 0.48 | 3.30 | 0.641 |  |  |  |  |
| Randomised into BRAVO | No | 23 / 145 | 15.9% | 10.6-22.5 |  |  |  |  |  |  |  |  |
|  | Randomised | 6 / 46 | 13.0% | 5.6-24.9 | 0.64 | 0.26 | 1.58 | 0.331 |  |  |  |  |
| Hospital type | Cancer centre | 13 / 66 | 19.7% | 11.5-30.5 |  |  |  |  |  |  |  |  |
|  | District | 16 / 125 | 12.8% | 7.8-19.5 | 0.60 | 0.29 | 1.25 | 0.171 |  |  |  |  |
| Grade | 2 | 5 / 40 | 12.5% | 4.9-25.2 |  |  |  |  |  |  |  |  |
|  | 3 | 21 / 143 | 14.7% | 9.6-21.2 | 1.11 | 0.42 | 2.95 | 0.836 |  |  |  |  |
| Background urothelium | Normal | 3 / 32 | 9.4% | 2.7-23 |  |  |  |  |  |  |  |  |
|  | Dysplasia | 0 / 10 | 0.0% | - |  |  |  |  |  |  |  |  |
|  | CIS | 15 / 74 | 20.3% | 12.4-30.5 | 2.04 | 0.59 | 7.05 | 0.26 |  |  |  |  |
| Stage | pTis | 3 / 7 | 42.9% | 13.9-76.5 |  |  |  |  |  |  |  |  |
|  | pTa | 11 / 98 | 11.2% | 6.1-18.6 |  |  |  |  |  |  |  |  |
|  | pT1 | 15 / 81 | 18.5% | 11.2-28 |  |  |  |  |  |  |  |  |
|  | pT2 | 0 / 3 | 0.0% | - |  |  |  |  |  |  |  |  |
|  | pTx | 0 / 2 | 0.0% | - | 0.23 | 0.06 | 0.82 | **0.024** | 3.68 | 0.72 | 18.78 | 0.117 |
| Growth | Papillary | 17 / 122 | 13.9% | 8.7-20.9 |  |  |  |  |  |  |  |  |
|  | Mixed | 8 / 40 | 20.0% | 9.9-34.2 |  |  |  |  |  |  |  |  |
|  | Solid | 0 / 7 | 0.0% | - | 1.39 | 0.60 | 3.23 | 0.442 |  |  |  |  |
| Histologic subtype | No | 27 / 173 | 15.6% | 10.8-21.6 |  |  |  |  |  |  |  |  |
|  | Yes | 2 / 18 | 11.1% | 2.4-31.1 | 0.69 | 0.16 | 2.89 | 0.608 |  |  |  |  |
| Re-resection | No | 8 / 60 | 13.3% | 6.5-23.6 |  |  |  |  |  |  |  |  |
|  | Yes | 21 / 131 | 16.0% | 10.5-23 | 1.08 | 0.48 | 2.44 | 0.855 |  |  |  |  |
| First treatment | BCG | 18 / 106 | 17.0% | 10.8-25 |  |  |  |  |  |  |  |  |
|  | HIVEC | 1 / 5 | 20.0% | 2.3-62.9 |  |  |  |  |  |  |  |  |
|  | RC | 2 / 43 | 4.7% | 1-14.1 |  |  |  |  |  |  |  |  |
|  | Other | 8 / 37 | 21.6% | 10.8-36.7 | 0.22 | 0.05 | 0.95 | **0.043** | 0.14 | 0.02 | 1.22 | 0.075 |

**Supplementary Table 5: Progression to more advanced disease within the entire cohort.**

|  |  | Metastases |  | | Univariable Cox | | |  | Multivariable Cox | | |  |
| --- | --- | --- | --- | --- | --- | --- | --- | --- | --- | --- | --- | --- |
|  |  | n / total | % | 95% CI | HR | Lower | Upper | p | HR | Lower | Upper | p |
| Age (years) | 0-70 | 3 / 77 | 4% | 1.1-10 |  |  |  |  |  |  |  |  |
|  | ≥71 | 16 / 115 | 14% | 8.5-21.1 | 4.36 | 1.27 | 15.00 | **0.019** | 2.03 | 0.47 | 8.72 | 0.343 |
| Sex | Female | 5 / 39 | 13% | 5.1-25.8 |  |  |  |  |  |  |  |  |
|  | Male | 14 / 153 | 9% | 5.3-14.5 | 0.69 | 0.25 | 1.91 | 0.474 |  |  |  |  |
| Randomised into BRAVO | No | 14 / 146 | 10% | 5.6-15.2 |  |  |  |  |  |  |  |  |
|  | Randomised | 5 / 46 | 11% | 4.3-22.2 | 0.91 | 0.33 | 2.52 | 0.848 |  |  |  |  |
| Hospital type | Cancer centre | 8 / 67 | 12% | 5.8-21.3 |  |  |  |  |  |  |  |  |
|  | District | 11 / 125 | 9% | 4.8-14.7 | 0.68 | 0.27 | 1.69 | 0.404 |  |  |  |  |
| Grade | 2 | 2 / 41 | 5% | 1-14.7 |  |  |  |  |  |  |  |  |
|  | 3 | 15 / 143 | 11% | 6.3-16.3 | 2.13 | 0.49 | 9.30 | 0.316 |  |  |  |  |
| Background urothelium | Normal | 4 / 32 | 13% | 4.4-27 |  |  |  |  |  |  |  |  |
|  | Dysplasia | 0 / 10 | 0% | - |  |  |  |  |  |  |  |  |
|  | CIS | 12 / 73 | 16% | 9.3-26.2 | 1.18 | 0.38 | 3.65 | 0.779 |  |  |  |  |
| Stage | pTis | 2 / 7 | 29% | 6.5-64.8 |  |  |  |  |  |  |  |  |
|  | pTa | 3 / 100 | 3% | 0.9-7.8 |  |  |  |  |  |  |  |  |
|  | pT1 | 14 / 80 | 18% | 10.4-26.9 |  |  |  |  |  |  |  |  |
|  | pT2 | 0 / 3 | 0% | - |  |  |  |  |  |  |  |  |
|  | pTx | 0 / 2 | 0% | - | 10.09 | 1.65 | 61.60 | **0.012** | 8.02 | 1.22 | 52.96 | **0.031** |
| Growth | Papillary | 9 / 123 | 7% | 4.7-12.9 |  |  |  |  |  |  |  |  |
|  | Mixed | 7 / 40 | 18% | 8.2-31.3 |  |  |  |  |  |  |  |  |
|  | Solid | 0 / 7 | 0% | - | 2.30 | 0.86 | 6.18 | 0.099 |  |  |  |  |
| Histologic subtype | No | 17 / 174 | 10% | 6-14.8 |  |  |  |  |  |  |  |  |
|  | Yes | 2 / 18 | 11% | 2.4-31.1 | 1.12 | 0.26 | 4.88 | 0.877 |  |  |  |  |
| Re-resection | No | 6 / 61 | 10% | 4.2-19.2 |  |  |  |  |  |  |  |  |
|  | Yes | 13 / 131 | 10% | 5.7-15.9 | 0.92 | 0.35 | 2.41 | 0.858 |  |  |  |  |
| First treatment | BCG | 13 / 106 | 12% | 7.1-19.5 |  |  |  |  |  |  |  |  |
|  | HIVEC | 0 / 7 | 0% | - |  |  |  |  |  |  |  |  |
|  | RC | 2 / 43 | 5% | 1.0-14.1 |  |  |  |  |  |  |  |  |
|  | Other | 4 / 36 | 11% | 3.9-24.3 | 0.33 | 0.07 | 1.47 | 0.146 |  |  |  |  |

**Supplementary Table 6: Development of metastases within the entire cohort.**

|  |  | Bladder Cancer Mortality | | | Univariable Cox | | | Multivariable Cox | | |
| --- | --- | --- | --- | --- | --- | --- | --- | --- | --- | --- |
|  |  | n / total | % | 95% CI | HR | 95% CI | p | HR | 95% CI | p |
| Age (years) | 0-70 | 2 / 77 | 2.6% | 0.5-8.1 |  |  |  |  |  |  |
|  | ≥71 | 15 / 116 | 12.9% | 7.8-19.9 | 5.33 | 1.21-23.48 | **0.027** | 4.87 | 1.1-21.58 | **0.037** |
| Sex | Female | 6 / 39 | 15.4% | 6.7-29.0 |  |  |  |  |  |  |
|  | Male | 11 / 154 | 7.1% | 3.9-12.0 | 0.41 | 0.15-1.13 | 0.086 |  |  |  |
| Randomised into BRAVO | No | 15 / 147 | 10.2% | 6.1-15.9 |  |  |  |  |  |  |
|  | Randomised | 2 / 46 | 4.3% | 0.9-13.2 | 0.18 | 0.02-1.38 | 0.099 |  |  |  |
| Hospital type | Cancer centre | 6 / 68 | 8.8% | 4.1-21.9 |  |  |  |  |  |  |
|  | District | 10 / 125 | 8.0% | 4.4-14.1 | 0.83 | 0.3-2.27 | 0.71 |  |  |  |
| Grade | 2 | 2 / 41 | 4.9% | 1.0-14.7 |  |  |  |  |  |  |
|  | 3 | 13 / 144 | 9.0% | 5.2-14.5 | 1.88 | 0.42-8.33 | 0.406 |  |  |  |
| Background urothelium | Normal | 3 / 32 | 9.4% | 2.8-23.6 |  |  |  |  |  |  |
|  | Dysplasia | 0 / 10 | 0.0% | . |  |  |  |  |  |  |
|  | CIS | 9/74 | 12.2% | 6.53-21.53 | 1.24 | 0.34-4.57 | 0.749 |  |  |  |
| Stage | Tis | 2 / 7 | 28.6% | 6.5-64.8 |  |  |  |  |  |  |
|  | Ta | 2 / 100 | 2.0% | 0.4-6.3 |  |  |  |  |  |  |
|  | T1 | 12 / 81 | 14.8% | 8.4-23.7 |  |  |  |  |  |  |
|  | T2 | 0 / 3 | 0.0% | . |  |  |  |  |  |  |
|  | Tx | 1 / 2 | 50.0% | 6.1-93.9 | 2.52 | 1.33-4.76 | **0.004** | 2.26 | 1.23-4.16 | **0.008** |
| Growth | Papillary | 8 / 124 | 6.5% | 3.3-12.2 |  |  |  |  |  |  |
|  | Mixed | 4 / 40 | 10.0% | 4.0-23.1 |  |  |  |  |  |  |
|  | Solid | 1 /7 | 14.3% | 2.5-51.3 | 1.50 | 0.64-3.55 | 0.351 |  |  |  |
| Histologic subtype | No | 15 / 175 | 8.6% | 5.1-13.4 |  |  |  |  |  |  |
|  | Yes | 2 / 18 | 11.1% | 2.4-31.1 | 1.48 | 0.34-6.53 | 0.603 |  |  |  |
| Re-resection | No | 5 / 62 | 8.1% | 3.1-16.8 |  |  |  |  |  |  |
|  | Yes | 12 / 131 | 9.2% | 5.1-15.0 | 0.95 | 0.33-2.73 | 0.92 |  |  |  |
| First treatment | BCG | 10 / 106 | 9.4% | 5.0-16.1 |  |  |  |  |  |  |
|  | HIVEC | 0 / 7 | 0.0% | . |  |  |  |  |  |  |
|  | RC | 2 / 43 | 4.7% | 1.0-14.1 |  |  |  |  |  |  |
|  | Other | 5 / 37 | 13.5% | 5.3-27.1 | 1.15 | 0.77-1.71 | 0.49 |  |  |  |

**Supplementary Table 7: Bladder cancer specific mortality within the cohort.** BCG - Bacillus Calmette–Guérin; HIVEC – hyperthermic intravesical MMC; CIS – carcinoma-in-situ; RC – radical cystectomy
